# Psychiatric and somatic morbidity patterns among patients diagnosed with anorexia nervosa and the risk of involuntary treatment: register-based cohort study

**DOI:** 10.1192/bjp.2025.4

**Published:** 2026-03

**Authors:** Line Bager, Hannah Chatwin, Katrine Holde, Birgitte Dige Semark, Mohamed Abdulkadir, Benjamin Mac Donald, Loa Clausen, Liselotte Vogdrup Petersen

**Affiliations:** National Centre for Register-Based Research, Aarhus University, Aarhus, Denmark; Department of Child and Adolescent Psychiatry, Aarhus University Hospital Psychiatry, Aarhus, Denmark; Department of Clinical Medicine, Aarhus University, Aarhus, Denmark; CIRRAU, Centre for Integrated Register-Based Research, Aarhus University, Denmark

**Keywords:** Anorexia nervosa, eating disorders, involuntary treatment, comorbidity, multimorbidity

## Abstract

**Background:**

Involuntary treatment for patients with anorexia nervosa is common and lifesaving, but also highly intrusive. Understanding how morbidity patterns relate to involuntary treatment can help minimise its use.

**Aim:**

We estimate the relative risk of involuntary treatment according to morbidity profiles in patients with anorexia nervosa.

**Method:**

This register-based cohort study included all individuals diagnosed with anorexia nervosa (ICD-10: F50.0, F50.1) between 1 January 2000 and 31 December 2016 in Denmark. Individuals were grouped by prior morbidities using latent class analysis (LCA). Cox proportional hazards regression estimated the relative risk of first involuntary treatment (e.g. involuntary admission, detention, locked wards) after a diagnosis with anorexia nervosa, regardless of the associated diagnosis. The relative risk of involuntary treatment was estimated with latent classes and the number of morbidities as exposure.

**Results:**

A total of 9892 individuals with anorexia nervosa were included (93.3% female), of which 821 (8.3%) individuals experienced at least one involuntary treatment event. The LCA produced six classes, with distinct morbidity profiles. The highest hazard ratio was observed for a group characterised by personality disorders, self-harm and substance misuse (hazard ratio 4.46, 95% CI: 3.43–5.79) followed by a high burden group with somatic and psychiatric disorders (hazard ratio 3.96, 95% CI: 2.81–5.59) and a group with developmental and behavioural disorders (hazard ratio 3.61, 95% CI: 2.54–5.11). The relative risk of involuntary treatment increased primarily with the number of psychiatric morbidities.

**Conclusions:**

Specific morbidity groups are associated with highly elevated risk of involuntary treatment among patients with anorexia nervosa. Targeting preventive interventions to high-risk groups may help reduce the need for involuntary treatment.

Individuals with anorexia nervosa are characterised by an intense fear of weight-gain, a disturbed body image and behaviours to achieve and maintain a low body-weight, such as restrictive dieting, purging and excessive exercise.^1^ Treatment drop out is frequent and relapse rates may reach as high as 31%.^2^ When individuals refuse voluntary treatment, involuntary treatment such as involuntary admission, detention, locked wards or tube feeding may be necessary. The legislative context in which involuntary treatment is implemented varies globally.^3^ In the Danish context, involuntary treatment can be implemented in psychiatric and somatic care, although all involuntary treatment beyond involuntary admission is only mandated in psychiatric care.^4^ Involuntary treatment is justified when an individual is assessed to be experiencing a severe mental impairment and it is deemed necessary, because of either the need for treatment or a perceived risk of danger to themselves or others, such as in cases of psychosis or similar conditions. Involuntary treatment may only be implemented after exhausting all possibilities of voluntary care.^4,5^ Any treatment against the patient’s will is registered as involuntary treatment and involuntary treatment is governed by principles that the least intrusive measures should be implemented first and that treatment should be proportional to the treatment aim.^6^ Despite these principles, implementation practices may vary.^6^ When implemented, involuntary treatment is often perceived negatively by patients, relatives and providers.^3,7^ For the patients, involuntary treatment may be experienced as stigmatising, intrusive, stressful or traumatic.^8,9^ Moreover, involuntary treatment may run the risk of exacerbating a hesitancy or reluctance to seek treatment and potentially undermine trust in healthcare providers.^10–12^

## Involuntary treatment in patients with anorexia nervosa

Previous research has shown that patients with anorexia nervosa are at high risk of experiencing involuntary treatment.^13^ It has been estimated that between 13% and 44% of severely ill in-patients with anorexia nervosa refuse treatment and therefore undergo involuntary treatment.^13^ In Denmark, 18% of in-patients with anorexia nervosa were subject to at least one involuntary treatment event^14^ and, in another study, a few patients (1%) accounted for the majority (67%) of the total involuntary treatment events.^15^ These involuntary treatment events were related not only to admissions with anorexia nervosa but also to admissions with other psychiatric disorders. They found that tube feeding^14^ and mechanical restraint^15^ were frequently used involuntary treatment measures for patients with anorexia nervosa. Risk factors for involuntary treatment among patients with anorexia nervosa include lower age, female gender, previous exposure to involuntary treatment and psychiatric and somatic morbidity.^13–15^ The implementation of involuntary treatment in the care of patients with anorexia nervosa cannot be reduced to their eating disorder alone but rather is a consequence of patients’ complex lives and illness history.^13,16^

## Morbidities in patients with anorexia nervosa

Danish population-based studies investigating prior and subsequent diagnoses have shown that individuals with prior psychiatric disorders are at increased risk of developing anorexia nervosa (median hazard ratio; 2.66, range 1.21–5.31) and those with a prior anorexia nervosa diagnosis are also at increased risk of being diagnosed with other psychiatric disorders (median hazard ratio; 3.80, range 2.48–6.15).^17^ Similarly, individuals with general medical conditions have increased risk of being diagnosed with anorexia nervosa, ranging from a hazard ratio (95% CI) of 1.08 (1.01–1.16) for prior diagnosed congenital disorders to 1.82 (1.58–2.10) for prior diagnosed circulatory disorders. In addition, those with a prior anorexia nervosa diagnosis are at increased risk of several medical conditions, with hazard ratios ranging from 1.27 (1.19–1.37) for later respiratory diseases to 2.22 (2.01–2.45) for circulatory disease.^18^ Morbidity in patients with anorexia nervosa is therefore common and likely influences the risk of involuntary treatment.

## Aim of the current study

Involuntary treatment remains an under-discussed and under-researched topic from an empirical point of view, despite being a longstanding ethical issue in mental health care.^19,20^ More knowledge is needed to understand what factors are associated with increased involuntary treatment in high-risk populations, such as patients with anorexia nervosa, to strengthen preventive efforts.^21^ The present study focuses on the burden and complexity of morbidities in patients with anorexia nervosa and their association with involuntary treatment. We hypothesise that a higher number of total psychiatric and somatic morbidities will be associated with an increased risk of involuntary treatment following an anorexia nervosa diagnosis, related to anorexia nervosa or readmissions among patients with anorexia nervosa for other disorders. We further hypothesise that patients characterised by specific groups of morbidities will exhibit distinct associations with involuntary treatment risk.

## Method

### Study design, participants and follow-up

This nationwide register-based cohort study spanned the study period from 1 January 2000 to 31 December 2016. The Danish registers include all individuals residing in Denmark from 1968 onwards. Through the Civil Registration System (CRS) it is possible to link information across registries, including medical databases. We included all individuals diagnosed with anorexia nervosa, that is, anorexia nervosa or atypical anorexia nervosa (ICD-10: F50.0, F50.1),^1^ in the study period. The index date of anorexia nervosa was determined as the first hospital-based discharge diagnosis at age six or later, registered either in the Danish National Patient Register (DNPR)^22^ or the Danish Psychiatric Central Research Register (DPCRR).^23^ Diagnoses are registered as ICD-8 codes before 1994.

### Outcome

The outcome was the first instance of involuntary treatment following a diagnosis with anorexia nervosa. The involuntary treatment event could be associated with an admission including a recorded anorexia nervosa diagnosis or any other disorder. Information on involuntary treatment came from the Registry of Coercive Measures in Psychiatric Treatment. This register was established in 1999 but did not have complete information until 2000.^24^ involuntary treatment is registered with a date and type referring to the following involuntary measures: admission, detention, locked wards, mechanical restraint, physical restraint, constant observation, medication, sedative medication, nasogastric tube feeding, somatic and electroconvulsive therapy (ECT).

### Exposures

The exposures were psychiatric and somatic morbidities including self-harm. Morbidities were operationalised in two ways: (a) by common groups of morbidities diagnosed before the anorexia nervosa diagnosis and (b) by the number of comorbid conditions over the lifetime. The ICD groups of somatic and psychiatric conditions were chosen based on previous research indicating that these conditions are prevalent among patients with eating disorders and that we hypothesised could influence the risk of involuntary treatment.^17,18,25^ We focused on 10 somatic groups (ICD-10): neoplasms (C00–C97, D00–D48), haematological (D50–D89), endocrine (E00–E90), neurological (G00–G99), circulatory (I00–I99), respiratory (J00–J99), gastrointestinal (K00–K93), dermatological (L00–L99), musculoskeletal (M00–M99) and genitourinary (N00–N99) (see Table S1 in the supplementary material for details, available at https://doi.org/10.1192/bjp.2025.4). We also included 10 psychiatric conditions (ICD-10): organic mental disorders (F00–09), substance use disorders (SUDs; F10–19), schizophrenia spectrum disorders (F20–29), mood disorders (F30–39), anxiety disorders (F40–48), other eating disorders (OED) (F50.2, F50.3, F50.8, F50.9), specific personality disorders (F60), intellectual disabilities (F70–79), developmental disorders (F84) and behavioural/emotional disorders (F90–98) (see Table S2 in the supplementary material for details of the full ICD-10 chapter title).

Finally, previous work has shown a high rate of suicide attempts^26^ and self-harm^27,28^ among those with eating disorders as well as an association between these and the subsequent need for involuntary treatment. In this study we included self-harm and possible suicide attempts as one comorbidity category, as we have no information on intent (see the supplementary material for the definition of self-harm).^29^ Information on the different exposure categories came from the DNPR and DPCRR and we counted the first diagnosis of each category. We utilised the work done by Pedersen et al^30^ to map ICD-10 codes to the corresponding ICD-8 codes designed for the Danish implementation of the ICD system.

### Covariates

Information on several covariates was collected as they were presupposed to be confounding the association between morbidities and involuntary treatment.^15,16^ These included patients’ gender, year of birth, age at the time of diagnosis, education, urbanicity and prior involuntary treatment. Education was defined as the highest attained education for the patient if the patient was 25 or older at the time of diagnosis, and otherwise the highest recorded education of either parent. Missing education was imputed with multivariate imputation.^31,32^ Urbanicity was categorised as follows: capital, suburb of capital, provincial city (municipalities with more than 100 000 inhabitants), provincial town (municipalities with between 10 000 and 100 000 inhabitants) and rural area (municipalities with towns with fewer than 10 000 inhabitants). Prior involuntary treatment was defined as an indicator variable for whether the individual had ever been exposed to an involuntary treatment event before the index diagnosis of anorexia nervosa. Information on these variables came from either the CRS, DNPR, DPCCR, the Danish Education Registers or the Registry of Coercive Measures in Psychiatric Treatment.^22–24^

### Statistical analysis

Frequencies and percentages were used to describe the characteristics of the population and a two-tailed Pearson’s χ^2^ or *t*-test was used to test difference between those with and without registered involuntary treatment. First, latent class analysis (LCA) was performed to group individuals based on their morbidity pattern. LCA is a probabilistic method, taking observed variables as input to estimate whether unobserved or latent groups exist within a population.^33^ The observed variables were the diagnosed somatic and psychiatric disorders. To avoid conditioning on the future, only morbidities diagnosed up to time of the anorexia nervosa diagnosis were included in the LCA. The number of classes was chosen after an evaluation of information criteria, class sizes and clinical relevance.^33^ Individuals were assigned to the class of highest probability of group membership. Second, the number of comorbid diagnoses was counted throughout the observation period as the first diagnosis in each diagnostic group within somatic and psychiatric diagnosis groups. Self-harm was included as a psychiatric morbidity.

Cox proportional hazards regressions were used to estimate the relative risk of involuntary treatment among patients diagnosed with anorexia nervosa. The study population was followed from time of the anorexia nervosa diagnosis up until the first involuntary treatment event, date of emigration, death or end of the study period (31 December 2016), whichever came first. Two analyses were conducted, one with the morbidity groups as the predictor and one with the number of somatic and psychiatric morbidities, with those without any registered morbidities as the reference. For the latter analysis, the number of morbidities was treated as time-varying and included in the Cox regression with an interaction between somatic and psychiatric morbidities. For both analyses, three models were analysed. Model 1 included time since diagnosis as the underlying timescale and adjusted for gender, birth year, age at anorexia nervosa diagnosis and urbanicity. The intermediate model (model S1) in addition adjusted for highest attained education at the time of the anorexia nervosa diagnosis (for the patient or the parents), while the last model (model 2) further added a variable indicating whether the individual had a registered involuntary treatment event before the anorexia nervosa diagnosis. All analyses and plots were generated using R version 4.1.3 for Windows (R Foundation; https://www.r-project.org/).

### Ethical approval

The study was registered with the Danish Data Protection Agency (2016-051-000001/745) and approved by Statistics Denmark (project ID: 703996) and the Danish Health Data Authority (SDS-ID: 1107). According to Danish law informed consent is not required for register-based studies.

## Results

Over the study period from 2000 to 2016, 9892 individuals were diagnosed with anorexia nervosa (93.3% female). Of those, 821 (8.3%) experienced at least one involuntary treatment event following their anorexia nervosa diagnosis. Table 1 provides descriptive statistics of the study population, showing that those exposed to involuntary treatment are slightly younger at the time of the anorexia nervosa diagnosis, with a mean (s.d.) age of 20 (10) years, compared to those unexposed, aged 21 (10) years. They also tend to live in smaller towns or rural areas, are more likely to have experienced involuntary treatment before their first anorexia nervosa diagnosis and have a higher number of prior morbidities.

**Table 1:** Characteristics for individuals diagnosed with anorexia nervosa and atypical anorexia nervosa between 2000 and 2016 by involuntary treatment status

Characteristic	Involuntary treatment (no), *N* = 9071^a^	Involuntary treatment (yes), *N* = 821^a^	Test statistic
Gender			χ^2^(1.0) = 2.6; *p* = 0.110
Female	8494 (94%)	757 (92%)	
Male	577 (6.4%)	64 (7.8%)	
Age at anorexia nervosa diagnosis	21 (10)	20 (10)	*t*(971) = 3.35; *p* < 0.001
Year of birth			χ²(3.0) = 3.8; *p* = 0.283
≤1960	246 (2.7%)	29 (3.5%)	
(1960, 1975]	670 (7.4%)	65 (7.9%)	
(1975, 1990]	3685 (41%)	311 (38%)	
>1990	4470 (49%)	416 (51%)	
Urbanicity			χ²(4.0) = 13; *p* = 0.011
Capital	1681 (19%)	131 (16%)	
Capital suburb	1420 (16%)	124 (15%)	
Provincial city	1298 (14%)	93 (11%)	
Provincial town	2334 (26%)	234 (29%)	
Rural area	2338 (26%)	239 (29%)	
SES: educational level			χ²(3.0) = 1.7; *p* = 0.635
Basic level	1109 (11%)	111 (13%)	
Short	3691 (40%)	339 (40%)	
Medium	2901 (32%)	254 (31%)	
High	1370 (15%)	117 (14%)	
(Missing)	180 (2%)	14 (2%)	
Involuntary treatment before a diagnosis with anorexia nervosa			χ²(1.0) = 532; *p* < 0.001
No	8998 (99%)	725 (88%)	
Yes	73 (0.8%)	96 (12%)	
Number of prior comorbidities			χ²(5.0) = 90; *p* < 0.001
0	1995 (22%)	135 (16%)	
1	2153 (24%)	164 (20%)	
2	1691 (19%)	131 (16%)	
3	1177 (13%)	102 (12%)	
4	753 (8.3%)	72 (8.8%)	
5+	1302 (14%)	217 (26%)	

SES, socioeconomic status.

a *n* (%); mean (s.d.); test statistics (degrees of freedom).

**Fig. 1:**
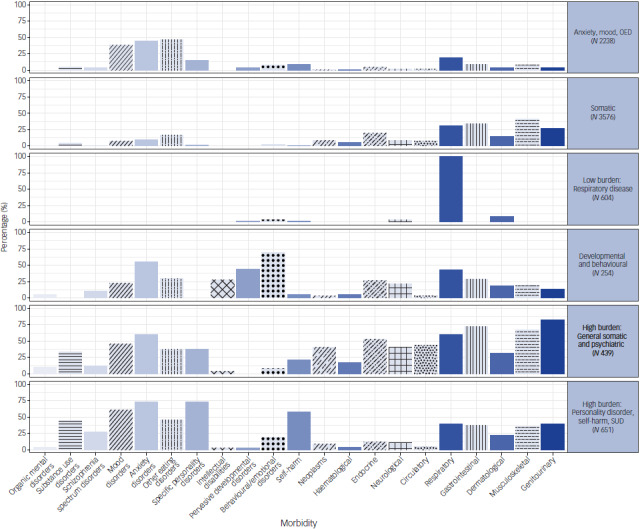
Morbidity classes for anorexia nervosa patients produced by the latent class analysis. OED, other eating disorders; SUD, substance use disorder.

The six-class LCA solution was selected, supported by the Bayesian information criterion and based on class size and clinical applicability (see Tables S3 and S4 for class fit statistics). Figure 1 illustrates the distribution of morbidities among the six classes. We labelled each group by its predominant diagnoses relative to the other groups, to illustrate both what characterises the group and how it differs from the other classes.

Table S5 provides a more detailed overview of the class characteristics, along with the group with no morbidities. Class one (*N* = 2238) is mainly characterised by anxiety, mood and OED with a mean (s.d.) age of 20 (7) years at the time of the anorexia nervosa diagnosis. Class two (*N* = 3576) is mainly characterised by a spectrum of somatic disorders and a mean (s.d.) age at diagnosis of 22 (10) years, while class three (*N* = 604) has a low burden of morbidities predominantly characterised by respiratory disorders and a mean (s.d.) age at diagnosis of 17 (4) years. Class four (*N* = 254) is distinct because of its relatively high prevalence of behavioural/emotional and pervasive developmental disorders, a relatively low mean (s.d.) age at diagnosis of 17 (5) years and with the highest proportion of males (23%). Both classes five and six are characterised by their high burden of morbidities. Class five (*N* = 439) is distinct because of having a high prevalence of both somatic and psychiatric disorders, with the bulk being among the somatic disorders. Class five also has the highest mean (s.d.) age at diagnosis of 38 (17) years. Class six (*N* = 651) differs because of having a high prevalence of personality disorders, self-harm and SUD. While these are more prevalent in this group compared to the others, this class is simultaneously characterised by a broad spectrum of psychiatric and somatic disorders. This group also has a relatively high mean (s.d.) age at first anorexia nervosa diagnosis, with 28 (11) years, and the lowest proportion of males (4.3%). The comparison group (*N* = 2130) with no morbidities is relatively young with a mean (s.d.) age of 17 (5) years, similar to classes three and four, and a proportion of males (5.0%) similar to classes five and six. Table S6 displays the mean time from morbidity diagnosis to a diagnosis with anorexia nervosa and the mean age at the time of the morbidity diagnosis.

**Fig. 2:**
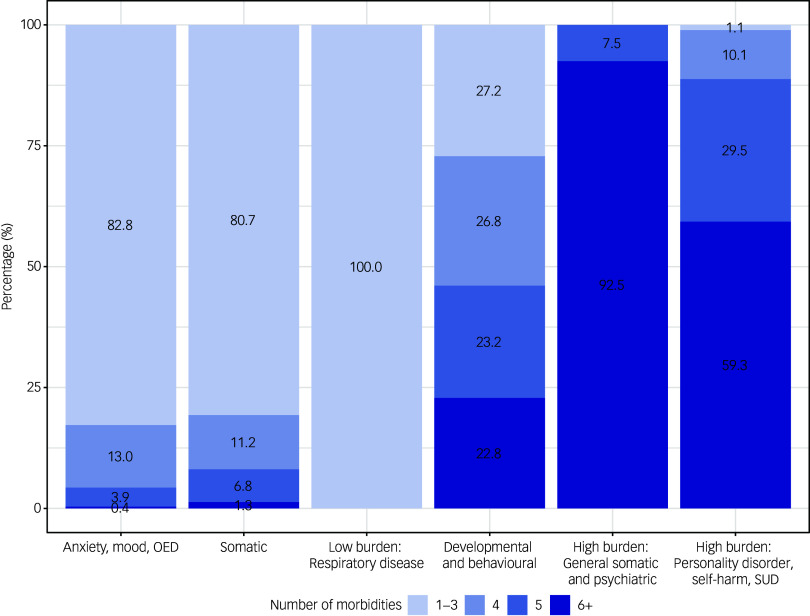
Number of morbidities per morbidity class. OED, other eating disorders; SUD, substance use disorder.

The burden of morbidities per class is illustrated in Fig. 2 as the number of morbidities per individual in each class. In the two high-burden groups, the majority (92.5% and 59.3%, respectively) of patients have six or more diagnosed morbidities at the time of their anorexia nervosa diagnosis, while in the low morbidity burden group (class three), all patients have one to three morbidities at the time of the anorexia nervosa diagnosis.

**Fig. 3:**
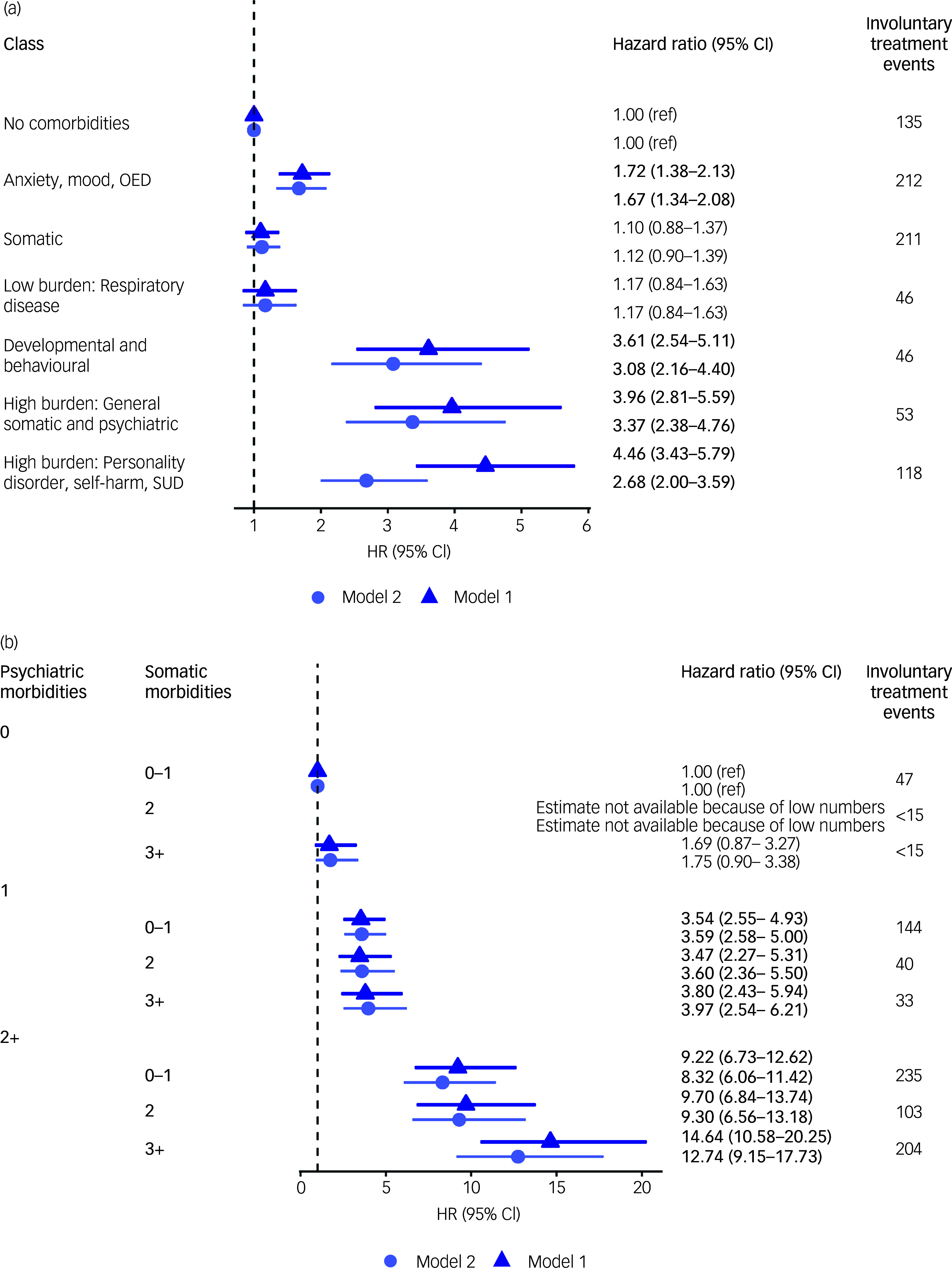
Risk of involuntary treatment for patients with anorexia nervosa by morbidity group (a) and the number of morbidities (b). HR, hazard ratio; OED, other eating disorders; SUD, substance use disorder. Model 1 is adjusted for gender, birth year, age at the time of the anorexia nervosa diagnosis and urbanicity. Model 2 is adjusted for model 1 covariates, as well as education and previous involuntary treatment.

The results from the regression with the latent classes as predictors of involuntary treatment are presented in Fig. 3(a). In model 1, the highest risk for an involuntary treatment event after the anorexia nervosa diagnosis was observed for the two high-morbidity groups characterised by personality disorder, self-harm and SUD (class six) and with general somatic and psychiatric disorders (class five) and the group with developmental and behavioural disorders. Class six was 4.5 times more likely to experience involuntary treatment compared to the reference group with no morbidities (hazard ratio 4.46, 95% CI: 3.43–5.79). Class five with general somatic and psychiatric disorders likewise had a highly elevated risk of involuntary treatment (hazard ratio 3.96, 95% CI: 1.17–2.46) compared to the reference group. Class four, characterised by developmental and behavioural disorders, did not have the same burden of morbidities as classes five and six (Fig. 2) but experienced a similarly elevated risk of involuntary treatment (hazard ratio 3.61, 95% CI: 2.54–5.11). Class one, characterised by anxiety, mood and OED, had a 72% (hazard ratio 1.72, 95% CI: 1.38–2.13) higher hazard for involuntary treatment compared to the reference group. The estimates for classes two and three characterised mainly by fewer and somatic disorders were not significantly different from patients with anorexia nervosa, but with no diagnosed morbidities. Model 2, with additional adjustment for education and previous involuntary treatment, yielded a substantial decrease in the risk for involuntary treatment in class six, characterised by personality disorder, self-harm and SUD (hazard ratio 2.68, 95% CI: 2.00–3.59).

Figure 3(b) displays the results from the regression with the number of somatic and psychiatric morbidities as the predictor of first involuntary treatment. The hazard ratios are increasing primarily with the number of psychiatric disorders. For both models 1 and 2, the highest relative hazards are found for the group with two or more psychiatric disorders and three or more somatic disorders. The group only characterised by somatic morbidities, in addition to their anorexia nervosa diagnosis, did not have a higher risk of involuntary treatment compared to the group with an anorexia nervosa diagnosis only. Similar to the analysis of the morbidity groups, the inclusion of previous involuntary treatment alters the estimates by attenuating the association between the number of morbidities and involuntary treatment in the high-risk groups. Model S1, adjusting for education in addition to model 1 covariates, did not alter the results significantly with either exposure (Tables S7 and S8 in the supplementary material).

## Discussion

This study investigated the risk of involuntary treatment after a diagnosis with anorexia nervosa by morbidity groups estimated with LCA and by the number of psychiatric and somatic disorders. We found that four out of six groups had a significantly elevated risk, relative to the reference with no other morbidities than anorexia nervosa. The highest overall risk was found for class six, which was the group with the highest prevalence of personality disorder, self-harm and SUD, but also schizophrenia spectrum disorders relative to the other groups. Those primarily characterised by somatic conditions showed no differences in risk of involuntary treatment compared to the reference with an anorexia nervosa diagnosis only. This finding was also emphasised in the analysis of the number of morbidities where the increase in the number of psychiatric morbidities corresponded to larger increases in hazards than the number of somatic morbidities.

Our findings align with the sparse existing evidence evaluating involuntary treatment in patients with an eating disorder.^19,34^ In a population with serious mental illness, but not eating disorders, personality disorders were found to increase the risk of involuntary treatment.^35^ In the Danish context, a register study found that personality disorders, schizophrenia spectrum disorders and autism spectrum disorders (ASDs) increased the risk of involuntary treatment among patients with anorexia nervosa.^14^ Similar to our findings, where involuntary treatment before the anorexia nervosa diagnosis increased the risk of subsequent involuntary treatment, prior psychiatric admissions with either anorexia nervosa^36,37^ or other conditions^15^ have been related to involuntary treatment in patients with anorexia nervosa.

Overall, the results presented here indicate that patients with more complex psychiatric morbidity profiles are at greatest risk of involuntary treatment. The high risk of involuntary treatment experienced by the group characterised by self-harm is not surprising as self-harm, if taking a form where it poses significant danger to the person’s health, is acknowledged as a legitimate reason for employing involuntary treatment. Nearly 60% of individuals in this group also had six or more diagnosed morbidities indicating a complex treatment need, potentially exacerbating the risk of involuntary treatment in the context of treatment for other illnesses than anorexia nervosa, although potentially very related to anorexia nervosa.^3^

Worth noting is also the high relative risk of involuntary treatment among the group characterised by developmental and emotional/behavioural disorders, comparable to the two groups with the highest burden of morbidities. The treatment of anorexia nervosa and other conditions could be complicated in this group because of the potential rigidity of thinking as well as difficulties in the communication of feelings associated with some of the disorders included in this group.^38^ These include hyperkinetic disorders, conduct disorders and ASDs, among others. The three high-risk groups are broadly characterised by having psychiatric illnesses that are typically considered very fundamental to the personality and long-term if not chronic conditions. Consequently, they can be seen as a fundamental vulnerability to other mental health difficulties and could form the basis for recurrent admissions to hospital and possibly involuntary treatment, either in themselves or as a result of secondary problems. Thus, they may be seen as chronic or persistent disorders, contrary to depression and anxiety, which is often more intermittent. The same division and influence on treatment needs also characterises somatic disorders. However, most somatic disorders in young people will probably be characterised by being transient, which could explain the limited impact of the frequency of somatic disorders on the risk of involuntary treatment. This might be what is seen in class two, mainly characterised by somatic disorders, and class three, almost exclusively characterised by respiratory diseases, as no additional risk of involuntary treatment was seen compared to those with no diagnosed morbidities.

For clinicians, these results emphasise the importance of complex morbidity for involuntary treatment among patients with anorexia nervosa, and hence the need to incorporate knowledge about patients’ full health profile and especially other psychiatric illnesses in addition to the eating disorder. The identified high-risk groups might benefit from targeted preventive interventions aimed at alleviating morbidities and reducing involuntary treatment risk.

### Strengths and limitations

There are several limitations of the study that should be kept in mind. Our study is limited to the information available from the registers. For instance, a variable such as self-harm relies on an approximation of several codes in the health registers and, as such, will entail a degree of imprecision. Moreover, we exclusively analyse hospital-based diagnoses; hence, individuals with undiagnosed anorexia nervosa or those treated exclusively in primary care will not be included in our study population. Similarly, some of the morbidities under investigation are, in less severe cases, only treated in primary care, such as hypertension, dyslipidaemia, depression and anxiety,^18^ and will therefore have no record in the registers used in this study.

Moreover, to avoid conditioning on the future, only morbidities before the anorexia nervosa diagnosis were included in the LCA. It should therefore be kept in mind that diagnoses given after the anorexia nervosa diagnosis were not included in the regression with the morbidity groups as predictors. While psychiatric and somatic diagnoses were included following the anorexia nervosa diagnosis in the regression of the number of diagnosed morbidities, none of the analyses could evaluate whether diagnosed morbidities were etiologically related to the anorexia nervosa diagnosis.^39^ Lastly, because of changes in recording practices, involuntary treatment is likely underreported for children under 15 before June 2015, in cases where a parent or guardian on behalf of the patients has consented to the treatment.^15^ While the data on involuntary treatment includes detailed records of types of involuntary treatment, in addition to involuntary admission, we were not able to analyse these separately or in subgroups as data became too sparse on stratification. Similarly, we do not know for which reason involuntary treatment was implemented, that is, whether it was directly related to anorexia nervosa or another concern.

Despite these limitations, this study has been able to comprehensively describe the risk of involuntary treatment in a large sample of patients with anorexia nervosa according to their morbidity profiles. The study utilised detailed data on involuntary treatment over 17 years for all individuals diagnosed with anorexia nervosa at a Danish hospital in the study period. Moreover, despite not being able to disaggregate the involuntary treatment measures used, information on involuntary treatment goes beyond that most typically analysed, namely involuntary admission. This data source is significant as information on involuntary treatment is not widely available or systematically collected in most countries.^40^ The quality of the data included was high, as all hospitals in Denmark by law are required to report involuntary treatment measures. Compared to previous studies where morbidities were included as associated pairs,^17,18^ we were able to include a comprehensive morbidity profile as a predictor. This approach adds a detailed overview of the complexity of the patients’ health profile and how that might affect the risk of involuntary treatment.

Overall, these results effectively illustrate the varied and complex disease profiles of patients with anorexia nervosa and the associated implications for the risk of experiencing involuntary treatment. Comorbid personality disorders, SUDs and self-harm, or developmental and behavioural/emotional disorders, add substantially to the risk of involuntary treatment, either on their own or together with anorexia nervosa. In addition, the more psychiatric disorders before or at the time of the first anorexia nervosa diagnosis, the greater the risk of subsequent involuntary treatment. The use of involuntary treatment is implemented as a last resort, with the safety and health of the patient (or sometimes others) in mind, but comes with potential significant cost for the patient’s experience and well-being.^5^ Continuous investigation of the circumstances around and risk factors for involuntary treatment should be prioritised to be able to understand and ultimately reduce its usage.

## Supporting information

Bager et al. supplementary material Bager et al. supplementary material

## Data Availability

Access to individual-level Danish data is governed by Danish authorities and can only be granted with prior approval. The governing bodies include the Danish Data Protection Agency, the Danish Health Data Authority, the Ethical Committee and Statistics Denmark. Researchers at Danish research institutions may obtain the relevant approval and data.

## Supplementary material

The supplementary material for this article can be found at https://doi.org/10.1192/bjp.2025.4
