# Supplementary material for: Psychiatric and somatic morbidity patterns among patients diagnosed with anorexia nervosa and the risk of involuntary treatment: register-based cohort study
Source: Br J Psychiatry. 2025 Apr 4;228(3):211–9. doi: 10.1192/bjp.2025.4 (PMC12912873; doi:10.1192/bjp.2025.4)
Supplement: Bager et al. supplementary material [file S0007125025000042sup001.docx]

**Title: Psychiatric and somatic morbidity patterns among patients diagnosed with anorexia nervosa and the risk of involuntary treatment: A register-based cohort study**

**Authors:**

Line Bager^a^, PhD., 0000-0003-2184-2808

Hannah Chatwin^a^, PhD., 0000-0001-7248-8568

Katrine Holde^a^, MSc., 0009-0004-0232-855X

Birgitte Dige Semark^a^, MSc., 0000-0002-1250-7066

Mohamed Abdulkadir^a^, PhD., 0000-0002-6080-257X

Benjamin Mac Donald, PhD., 0000-0002-7901-3642

*Loa Clausen^b,c^, PhD., 0000-0002-4559-8347

*Liselotte Vogdrup Petersen^a,d^, PhD., 0000-0002-0479-5379

**Affiliations:**

^a^NCRR, National Centre for Register-Based Research, Aarhus University, Aarhus, Denmark

^b^Department of Clinical Medicine, Aarhus University, Aarhus, Denmark

^c^Department of Child and Adolescent Psychiatry, Aarhus University Hospital Psychiatry, Aarhus, Denmark

^d^CIRRAU, Centre for Integrated Register-based Research, Aarhus University, Denmark

*Last author.

**Corresponding author:** Line Bager, The National Centre for Register-based Research, Aarhus University, Fuglesangs Allé 26, 8210 Aarhus, Denmark, [lbager.ncrr@au.dk](mailto:lbager.ncrr@au.dk)

Contents

[Table S1: ICD-10 codes for somatic morbidities*. 3](#_Toc182570928)

[Table S2: ICD-10 codes for psychiatric morbidities*. 4](#_Toc182570929)

[Definition of self-harm based on Danish registry data ^1,2^ 5](#_Toc182570930)

[Table S3: Model fit statistics for evaluating number latent classes 5](#_Toc182570931)

[Table S4: Median probability of class membership (6 class solution) by the Latent Class Analysis 5](#_Toc182570932)

[Table S5: Anorexia nervosa and atypical anorexia (AN) patients’ characteristics per comorbidity class* 6](#_Toc182570933)

[Table S6: Timing of the morbidity diagnosis prior to a diagnosis with anorexia nervosa 8](#_Toc182570934)

[Table S7: Regression results – hazard ratios (HR) for involuntary treatment (IT) with morbidity classes as a predictor* 9](#_Toc182570935)

[Table S8: Regression results – hazard ratios (HR) for involuntary treatment (IT) with number of comorbidities as a predictor* 9](#_Toc182570936)

## Table S1: ICD-10 codes for somatic morbidities*.

| Abbreviated heading | ICD-10 chapter title | ICD-10 code |
| --- | --- | --- |
| Neoplasms | Neoplasms | C00-C97, D00-D48 |
| Haematological | Diseases of the blood and blood-forming organs and certain disorders involving the immune mechanism | D50-D89 |
| Endocrine | Endocrine, nutritional and metabolic diseases | E00-E90 |
| Neurological | Diseases of the nervous system | G00-G99 |
| Circulatory | Diseases of the circulatory system | I00-I99 |
| Respiratory | Diseases of the respiratory system | J00-J99 |
| Gastrointestinal | Diseases of the digestive system | K00-K93 |
| Dermatological | Diseases of the skin and subcutaneous tissue | L00-L99 |
| Musculoskeletal | Diseases of the musculoskeletal system and connective tissue | M00-M99 |
| Genitourinary | Diseases of the genitourinary system | N00-N99 |

**^*^**Excluded diagnoses (ICD-10): Certain infectious and parasitic diseases (A00-B99), diseases of the eye and adnexa (H00-H59), pregnancy, childbirth and the puerperium (O00-O99), certain conditions originating in the perinatal period (P00-P96), congenital malformations, deformations and chromosomal abnormalities (Q00-Q99), symptoms, signs and abnormal clinical and laboratory findings, not elsewhere classified (R00-R99), factors influencing health status and contact with health services (Z00-Z99), codes for special purposes (U00-U85). See methods section for which injuries and external causes of morbidity are included.

## Table S2: ICD-10 codes for psychiatric morbidities*.

| Abbreviated heading | ICD-10 chapter title | ICD-10 code | Min. age at diagnosis (years) |
| --- | --- | --- | --- |
| Organic mental disorders | Organic, including symptomatic, mental disorders | F00-F09 | 1 |
| Substance use disorders | Mental and behavioural disorders due to psychoactive substance use | F10-F19 | 10 |
| Schizophrenia spectrum disorders | Schizophrenia, schizotypal and delusional disorders | F20-F29 | 10 |
| Mood disorders | Mood [affective] disorders | F30-F39 | 6 |
| Anxiety disorders | Neurotic, stress-related and somatoform disorders | F40-F48 | 6 |
| Other eating disorders | Bulimia nervosa, atypical bulimia nervosa, other eating disorders, eating disorder, unspecified | F50.2, F50.3, F50.8, F50.9 | 6 |
| Specific personality disorders | Specific personality disorders | F60 | 10 |
| Intellectual disabilities | Mental retardation | F70-F79 | 1 |
| Developmental disorders | Pervasive developmental disorders | F84 | 1 |
| Behavioural/emotional disorders | Behavioural and emotional disorders with onset usually occurring in childhood and adolescence | F90-F98 | 1 |

**^*^**Excluded diagnoses (ICD-10): Overeating associated with other psychological disturbances (F50.4), vomiting associated with other psychological disturbances (F50.5), nonorganic sleep disorders (F51), sexual dysfunction, not caused by organic disorder or disease (F52), mental and behavioural disorders associated with the puerperium, not elsewhere classified (F53), psychological and behavioural factors associated with disorders or diseases classified elsewhere (F54), abuse of non-dependence-producing substances (F55), unspecified behavioural syndromes associated with physiological disturbances and physical factors (F59), mixed and other personality disorders (F61), enduring personality changes, not attributable to brain damage and disease (F62), habit and impulse disorders (F63), gender identity disorders (F64), disorders of sexual preference (F65), psychological and behavioural disorders associated with sexual development and orientation (F66), other disorders of adult personality and behaviour (F68), unspecified disorder of adult personality and behaviour (F69), specific developmental disorders of speech and language (F80), specific developmental disorders of scholastic skills (F81), specific developmental disorder of motor function (F82), mixed specific developmental disorders (F83), other disorders of psychological development (F88), unspecified disorder of psychological development (F89), unspecified mental disorder (F99-F99).

## Definition of self-harm based on Danish registry data ^1,2^

Self-harm was included if one of the following conditions were met;

1. the individual had a diagnosis of intentional self-harm (ICD-10: X60-X84, ICD-8: E950-E959),
2. if the cause of contact was registered as “suicide attempt”,
3. if the primary diagnosis of the contact was a psychiatric diagnosis (ICD-10: F00-99) and the auxiliary diagnosis was a wound to the lower arm, wrist, or hand (S51, S55, S59, S61, S65, S69),
4. the primary contact diagnosis was a psychiatric disorder and a secondary diagnosis of poisoning (T39, T42, T43, T58),
5. the main diagnosis was poisoning (T39, T42, T43, T58) if the patient was at least six years old.

## Table S3: Model fit statistics for evaluating number latent classes

| Model | Log likelihood | AIC | BIC | Entropy | Smallest class size (%) |
| --- | --- | --- | --- | --- | --- |
| Two classes | -57,009.80 | 114,105.6 | 114,404.7 | 0.7743967 | 15.89 |
| Three classes | -56,552.92 | 113,235.8 | 113,688.0 | 0.5979144 | 12.77 |
| Four classes | -56,349.92 | 112,873.8 | 113,479.1 | 0.6110937 | 6.58 |
| Five classes | -56,215.61 | 112,649.2 | 113,407.5 | 0.6164857 | 6.11 |
| Six classes | -56,088.56 | 112,439.1 | **113,350.5*** | 0.6209219 | 3.27 |
| Seven classes | -55,994.89 | 112,295.8 | 113,360.2 | 0.6145903 | 1.42 |
| Eight classes | -55,908.83 | 112,167.7 | 113,385.1 | **0.6258120*** | 1.35 |

*Preferred solution

## Table S4: Median probability of class membership (6 class solution) by the Latent Class Analysis

| Class | Median | IQR lower | IQR upper |
| --- | --- | --- | --- |
| 1 | 0.7016227 | 0.5635694 | 0.7739314 |
| 2 | 0.8138422 | 0.6572143 | 0.8706439 |
| 3 | 0.6916413 | 0.6916413 | 0.6916413 |
| 4 | 0.7274850 | 0.5408639 | 0.9063425 |
| 5 | 0.8135303 | 0.6310587 | 0.9467228 |
| 6 | 0.7499650 | 0.5781402 | 0.9209211 |

*Interquartile range

## Table S5: Anorexia nervosa and atypical anorexia (AN) patients’ characteristics per comorbidity class*

| **Characteristic** | **No comorbidities, N = 2,130^1^** | **Anxiety, mood, OED, N = 2,238^1^** | **Somatic, N = 3,576^1^** | **Low burden: Respiratory disease, N = 604^1^** | **Developmental and behavioural, N = 254^1^** | **High burden: General somatic and psychiatric, N = 439^1^** | **High burden: Personality disorder, self-harm, SUD, N = 651^1^** |  |
| --- | --- | --- | --- | --- | --- | --- | --- | --- |
| **Sex** |  |  |  |  |  |  |  |  |
| Female | 2,023 (95%) | 2,088 (93%) | 3,355 (94%) | 552 (91%) | 195 (77%) | 415 (95%) | 623 (96%) |  |
| Male | 107 (5.0%) | 150 (6.7%) | 221 (6.2%) | 52 (8.6%) | 59 (23%) | 24 (5.5%) | 28 (4.3%) |  |
| **Age at AN diagnosis** | 17 (5) | 20 (7) | 22 (10) | 17 (4) | 17 (5) | 38 (17) | 28 (11) |  |
| **Year of birth** |  |  |  |  |  |  |  |  |
| ≤1974 | 56 (2.6%) | 148 (6.6%) | 353 (9.9%) | 7 (1.2%) | 3 (1.2%) | 202 (46%) | 147 (23%) |  |
| 1975-1984 | 255 (12%) | 422 (19%) | 668 (19%) | 81 (13%) | 11 (4.3%) | 109 (25%) | 185 (28%) |  |
| 1985-1994 | 1,021 (48%) | 971 (43%) | 1,494 (42%) | 291 (48%) | 96 (38%) | 105 (24%) | 256 (39%) |  |
| ≥1995 | 798 (37%) | 697 (31%) | 1,061 (30%) | 225 (37%) | 144 (57%) | 23 (5.2%) | 63 (9.7%) |  |
| **Urbanicity** |  |  |  |  |  |  |  |  |
| Capital | 320 (15%) | 407 (18%) | 707 (20%) | 88 (15%) | 36 (14%) | 103 (23%) | 151 (23%) |  |
| Capital suburb | 307 (14%) | 328 (15%) | 550 (15%) | 114 (19%) | 43 (17%) | 83 (19%) | 119 (18%) |  |
| Provincial city | 366 (17%) | 325 (15%) | 484 (14%) | 54 (8.9%) | 15 (5.9%) | 52 (12%) | 95 (15%) |  |
| Provincial town | 546 (26%) | 585 (26%) | 921 (26%) | 164 (27%) | 85 (33%) | 99 (23%) | 168 (26%) |  |
| Rural area | 591 (28%) | 593 (26%) | 914 (26%) | 184 (30%) | 75 (30%) | 102 (23%) | 118 (18%) |  |
| **Educational level** |  |  |  |  |  |  |  |  |
| Basic level | 131 (6.2%) | 269 (12%) | 389 (11%) | 48 (7.9%) | 41 (16%) | 144 (33%) | 198 (30%) |  |
| Short | 825 (39%) | 946 (42%) | 1,480 (41%) | 235 (39%) | 98 (39%) | 181 (41%) | 265 (41%) |  |
| Medium | 732 (34%) | 714 (32%) | 1,177 (33%) | 219 (36%) | 81 (32%) | 85 (19%) | 147 (23%) |  |
| High | 442 (21%) | 309 (14%) | 530 (15%) | 102 (17%) | 34 (13%) | 29 (6.6%) | 41 (6.3%) |  |
| **Number of prior comorbidities** |  |  |  |  |  |  |  |  |
| 0 | 2,130 (100%) | 0 (0%) | 0 (0%) | 0 (0%) | 0 (0%) | 0 (0%) | 0 (0%) |  |
| 1-3 | 0 (0%) | 1,852 (83%) | 2,886 (81%) | 604 (100%) | 69 (27%) | 0 (0%) | 7 (1.1%) |  |
| 4 | 0 (0%) | 290 (13%) | 401 (11%) | 0 (0%) | 68 (27%) | 0 (0%) | 66 (10%) |  |
| 5 | 0 (0%) | 88 (3.9%) | 242 (6.8%) | 0 (0%) | 59 (23%) | 33 (7.5%) | 192 (29%) |  |
| 6+ | 0 (0%) | 8 (0.4%) | 47 (1.3%) | 0 (0%) | 58 (23%) | 406 (92%) | 386 (59%) |  |
| **Involuntary treatment after a diagnosis with AN** |  |  |  |  |  |  |  |  |
| No | 1,995 (94%) | 2,026 (91%) | 3,365 (94%) | 558 (92%) | 208 (82%) | 386 (88%) | 533 (82%) |  |
| Yes | 135 (6.3%) | 212 (9.5%) | 211 (5.9%) | 46 (7.6%) | 46 (18%) | 53 (12%) | 118 (18%) |  |
| **Mean follow-up time** | 7.2 (5.0) | 6.4 (4.7) | 6.4 (4.7) | 7.3 (5.1) | 4.1 (3.9) | 4.9 (4.2) | 5.7 (4.5) |  |
| ^1^n (%); Mean (SD)  *Involuntary treatment prior to the diagnosis of anorexia nervosa cannot be reported per comorbidity class due to small numbers in the low-risk groups. SES: Educational level reports the imputed values, due to low numbers of missing per group | | | | | | | | |

## Table S6: Timing of the morbidity diagnosis prior to a diagnosis with anorexia nervosa

| **Morbidity** | **Mean time from morbidity diagnosis to AN diagnosis (SD)** | **Mean age at morbidity diagnosis (SD)** |
| --- | --- | --- |
| Organic Mental Disorders | 8 (9) | 36 (21) |
| Substance Use Disorders | 6.2 (6.8) | 25 (10) |
| Schizophrenia | 6 (7) | 24 (10) |
| Mood Disorders | 3.5 (4.9) | 23 (10) |
| Anxiety Disorders | 4.3 (5.5) | 22 (9) |
| Other Eating Disorders | 1.90 (3.17) | 21 (8) |
| Specific Personality Disorders | 6 (7) | 24 (8) |
| Intellectual Disabilities | 6 (6) | 18 (9) |
| Pervasive Developmental Disorders | 3.3 (3.8) | 16.2 (5.9) |
| Behavioural/Emotional Disorders | 5.3 (5.6) | 17 (8) |
| Neoplasms | 8 (7) | 27 (14) |
| Diseases of the blood | 8 (8) | 26 (18) |
| Endocrine | 7 (7) | 23 (14) |
| Neurologic | 8 (7) | 24 (14) |
| Circulatory | 6.9 (6.4) | 29 (16) |
| Respiratory | 13 (7) | 13 (15) |
| Gastrointestinal | 9 (7) | 21 (13) |
| Skin | 9 (6) | 21 (13) |
| Musculoskeletal | 6.8 (5.6) | 22 (12) |
| Genitourinary | 8 (7) | 23 (11) |
| Self-harm | 5.9 (6.5) | 21 (8) |

## Table S7: Regression results – hazard ratios (HR) for involuntary treatment (IT) with morbidity classes as a predictor*

|  | **Model S1:** Adjusted for education | | |
| --- | --- | --- | --- |
| Classes | IT (yes) | HR | (95% CI) |
| No comorbidities | 135 | 1 | - |
| Anxiety, mood, OED | 212 | 1.73 | (1.40-2.15) |
| Somatic | 211 | 1.11 | (0.89-1.38) |
| Low burden: Respiratory disease | 46 | 1.18 | (0.84-1.64) |
| Developmental and behavioural | 46 | 3.67 | (2.59-5.22) |
| High burden: General somatic and psychiatric | 53 | 4.04 | (2.86-5.70) |
| High burden: Personality disorder, self-harm, SUD | 118 | 4.58 | (3.52-5.97) |

*Adjusted for sex, birth year, age at the time of the anorexia nervosa diagnosis, and urbanicity

## Table S8: Regression results – hazard ratios (HR) for involuntary treatment (IT) with number of comorbidities as a predictor*

|  | **Model S1:** Adjusted for education | | |
| --- | --- | --- | --- |
| Somatic x Psychiatric morbidities | IT (yes) | HR | (95% CI) |
| Somatic, 0-1; Psychiatric, 0 | 47 | 1 | - |
| Somatic, 2; Psychiatric, 0 | <15 | NA | NA |
| Somatic, 3+; Psychiatric, 0 | <15 | 1.71 | (0.88-3.30) |
| Somatic, 0-1; Psychiatric, 1 | 144 | 3.59 | (2.58-4.99) |
| Somatic, 2; Psychiatric, 1 | 40 | 3.54 | (2.32-5.42) |
| Somatic, 3+; Psychiatric, 1 | 33 | 3.91 | (2.50-6.11) |
| Somatic, 0-1; Psychiatric, 2+ | 235 | 9.43 | (6.88-12.92) |
| Somatic, 2; Psychiatric, 2+ | 103 | 10.04 | (7.08-14.24) |
| Somatic, 3+; Psychiatric, 2+ | 204 | 15.31 | (11.05-21.20) |

* Adjusted for sex, birth year, age at the time of the anorexia nervosa diagnosis, and urbanicity

NA: Estimate not available due to low numbers

References:

1. Gasse C, Danielsen AA, Pedersen MG, Pedersen CB, Mors O, Christensen J. Positive predictive value of a register-based algorithm using the Danish National Registries to identify suicidal events. *Pharmacoepidemiol Drug Saf*. 2018;27(10):1131-1138. doi:10.1002/pds.4433

2. Zerwas S, Larsen JT, Petersen L, Thornton LM, Mortensen PB, Bulik CM. The incidence of eating disorders in a Danish register study: Associations with suicide risk and mortality. *J Psychiatr Res*. 2015;65:16-22. doi:10.1016/j.jpsychires.2015.03.003
